# Retraction Note: HOXA7 plays a critical role in metastasis of liver cancer associated with activation of *Snail*

**DOI:** 10.1186/s12943-023-01855-2

**Published:** 2023-09-13

**Authors:** Bo Tang, Guangying Qi, Xiaoyu Sun, Fang Tang, Shengguang Yuan, Zhenran Wang, Xingsi Liang, Bo Li, Shuiping Yu, Jie Liu, Qi Huang, Yangchao Wei, Run Zhai, Biao Lei, Xinjin Guo, Songqing He

**Affiliations:** 1 Department of Hepatobiliary Surgery, Affiliated Hospital, Guilin Medical University, 541001 Guilin, Guangxi People’s Republic of China; 2 Laboratory of Liver Injury and Repair Molecular Medicine, Guilin Medical University, 541001 Guilin, Guangxi People’s Republic of China; 3 Department of Pathology and Physiopathology, Guilin Medical University, 541004 Guilin, Guangxi People’s Republic of China; 4 Department of Gastroenterology, First Affiliated Hospital of Dalian Medical University, 116011 Dalian, Liaoning People’s Republic of China; 5 Department of Clinical Pharmacology, College of Pharmacy, Dalian Medical University, 116011 Dalian, Liaoning People’s Republic of China

**Retraction Note:** ***Mol Cancer*** **15** **, 57 (2016)**

10.1186/s12943-016-0540-4

The Editor-in-Chief has retracted this article at the authors’ request. After publication, concerns were raised regarding multiple cases of H&E staining and western blot image overlap with other articles that were submitted and published within a close time frame [1–8].

The authors have stated that the data were mislabelled and the images were misused. The authors and the Editor-in-Chief therefore no longer have confidence in the presented data.

Bo Tang has stated on behalf of all co-authors that they agree to this retraction.
